# Association between *HOTAIR* lncRNA Polymorphisms and Coronary Artery Disease Susceptibility

**DOI:** 10.3390/jpm11050375

**Published:** 2021-05-04

**Authors:** In-Jai Kim, Jeong-Yong Lee, Hyeon-Woo Park, Han-Sung Park, Eun-Ju Ko, Jung-Hoon Sung, Nam-Keun Kim

**Affiliations:** 1 CHA Bundang Medical Center, Department of Cardiology, CHA University, Seongnam 13496, Korea; mdij24@chol.com (I.-J.K.); atropin5@cha.ac.kr (J.-H.S.); 2 Department of Biomedical Science, College of Life Science, CHA University, Seongnam 13488, Korea; smilee3625@naver.com (J.-Y.L.); aabb1114@naver.com (H.-W.P.); hahnsung@naver.com (H.-S.P.); ejko05@naver.com (E.-J.K.)

**Keywords:** single nucleotide variant, coronary artery disease, long non-coding RNA, HOX transcript antisense RNA

## Abstract

Coronary artery disease (CAD), one of the most frequent causes of mortality, is the most common type of cardiovascular disease. This condition is characterized by the accumulation of plaques in the coronary artery, leading to blockage of blood flow to the heart. The main symptom of CAD is chest pain caused by blockage of the coronary artery and shortness of breath. HOX transcript antisense RNA gene (*HOTAIR*) is a long non-coding RNA which is well-known as an oncogene involved in various cancers, such as lung, breast, colorectal, and gastric cancer. We selected six single nucleotide polymorphisms, rs4759314 A>G, rs1899663 G>T, rs920778 T>C, rs7958904 G>C, rs12826786 C>T, and rs874945 C>T, for genotype frequency analysis and assessed the frequency of *HOTAIR* gene polymorphisms in 442 CAD patients and 418 randomly selected control subjects. To analyze the differences between these two populations, we performed a Student’s *t*-test, adjusted odds ratio (AOR), 95% confidence intervals (CIs), and ANOVA analysis. According to our baseline characteristic analysis, control subjects and CAD patients were significantly different in hypertension and diabetes mellitus. We also found that the rs4759314 A>G, rs1899663 G>T, and rs12826786 C>T genotypes were strongly associated with CAD susceptibility (AA vs. AG+GG: AOR = 0.608, 95% CI = 0.393−0.940, *p* = 0.025; GG vs. TT: AOR = 2.276, 95% CI = 1.125−4.607, *p* = 0.022; CC vs. CT+TT: AOR = 1.366, 95% CI = 1.027−1.818, *p* = 0.032, respectively). Our data also demonstrated that the genotype of *HOTAIR* polymorphisms, genotype combination, and haplotype analysis affect disease occurrence. Moreover, these polymorphisms are linked to clinical factors that contribute to disease susceptibility. In conclusion, results from our study suggest that *HOTAIR* polymorphisms may be useful novel biomarkers for diagnosing CAD.

## 1. Introduction

The most common form of cardiovascular disease is coronary artery disease (CAD), in which a plaque builds up inside the coronary arteries, leading to a complete blockage of blood flow to the heart that can result in a heart attack and chest pain (angina). CAD is characterized by atherosclerosis in the epicardial coronary arteries. According to the World Health Organization, CAD is one of the most common causes of mortality [1], with classic risk factors that include age, gender, hypertension (HTN), diabetes mellitus (DM), and smoking [2,3].

Long non-coding RNAs (lncRNA) are known as a non-transcript RNA and characterized by a length greater than 200 nucleotides. The functions of lncRNA are not fully understood, but studies have suggested their involvement in regulating gene expression and post-transcriptional modification [4]. Moreover, lncRNA has been shown to play a role in various diseases, especially cancer and cardiovascular diseases, including myocardial infarction (MI), atrial fibrillation, and CAD [5,6,7,8,9,10,11,12].

Homeobox (HOX) transcript antisense RNA (*HOTAIR*) is a lncRNA located on chromosome 12q13.13 that is encoded in the *HOXC* gene cluster [13], which consists of 6232 nucleotides [9]. HOTAIR activates *HOXD* gene expression by decreasing the tri-methylation of histone H3K27 [9] and recruiting the polycomb repressive complex (PRC2). *HOTAIR* also regulates histone H3K4, thus promoting interactions with lysine-specific histone demethylase 1A (LSD1) [9]. *HOTAIR* has been reported to be a key regulator of cancer, including colorectal, prostate, gastric, and ovarian cancer [13,14]. Finally, *HOTAIR* overexpression suppressed cell viability and upregulated cell apoptosis by oxidized low-density lipoprotein (LDL) [15].

HOTAIR is frequently reported as an oncogene; however, studies have also associated it with cardiovascular diseases, such as CAD and heart failure. Previous reports demonstrated that HOTAIR acts as a negative regulator of MI in mice by absolving miR-519d-3p [16]. Moreover, HOTAIR was reported as an antagonist of cardiovascular disease that protects against hypoxia exposure or acute myocardial ischemia by absolving miR-1 and miR-125 to inhibit apoptosis and regulate downstream genes [17].

Single nucleotide polymorphisms (SNPs) are associated with many disease occurrences [18,19,20], and *HOTAIR* SNPs have been associated with recurrent implant failure and various cancers. However, the association between *HOTAIR* SNPs and CAD remains poorly understood. In this study, we analyzed the differences between CAD patients and healthy controls and found that the *HOTAIR* rs4759314 A>G, rs1899663 C>T, and rs12826786 C>T polymorphisms are genetically associated with the prevalence of CAD. 

## 2. Materials and Methods

### 2.1. Study Population 

In the South Korean provinces of Seoul and Gyeonggi-do, study subjects were recruited between 2006 and 2015 from the Department of Cardiology at the CHA Bundang Medical Center in Seongnam, South Korea. The study was approved by the Institutional Review Board of CHA Bundang Medical Center. (IRB number: 2013-10-114) In total, 442 patients diagnosed with CAD were referred from the Department of Cardiology, and all patients who presented with stable CAD or acute coronary syndromes (including unstable angina with or without ST-segment elevation) and at least one coronary lesion with >50% stenosis in a vessel with a diameter of 2.25–4.00 mm were screened for eligibility. No restrictions were placed on the total number of treated lesions, which vessels were treated, lesion length, or the number of stents implanted. Exclusion criteria included a history of acute myocardial infarction and life expectancy <1 year. All patients underwent coronary angiography and electrocardiography. Diagnoses were made by coronary angiography and confirmed by at least one independent experienced cardiologist.

During the same time period, 418 gender- and age-matched control subjects were selected from patients who visited the Department of Cardiology for health examinations, including biochemical testing, electrocardiograms, and coronary computed tomography scans. The control group was subjected to the same exclusion criteria as the CAD group, with the addition of reported recent history of anginal symptoms. Hypertension was defined as a systolic pressure > 140 mmHg and a diastolic pressure > 90 mmHg on more than one occasion and included patients who were treated with hypertensive medications during the study period. DM was defined as a fasting plasma glucose level > 126 mg/dL (7.0 mmol/L) and included patients taking diabetic medications. Smoking referred to patients who smoked at the time of the study. Hyperlipidemia was defined as a high fasting serum total cholesterol level (≥240 mg/dL) or a history of anti-hyperlipidemic treatment.

### 2.2. Genetic Analyses

Genomic DNA was extracted from whole blood samples using the G-DEX II Genomic DNA Extraction kit (Intron Biotechnology Inc., Seongnam, Korea). DNA was diluted to 100 ng/µL with 1× Tris-EDTA (TE) buffer, and then 1 µL of each sample was used to amplify the polymorphisms. 

All PCR experiments were performed using the AccuPower HotStart PCR PreMix (Bioneer Corporation, Daejeon, Korea). For the genotyping analysis, rs7958904, rs920778, and rs12826786 were analyzed using a Taqman genotyping assay (Applied Biosystems, Foster City, CA, USA), whereas rs1899663 and rs4759314 genotyping was performed using polymerase chain reaction-restriction fragment length polymorphism (PCR-RFLP) analysis. The following primer sets were used in this study: rs1899663, 5’- TTT TCC AGT TGA GGA GGG TGG A -3’ (forward primer) and 5’- CTA ATG GCA AGG GAA GGG AAG G -3’ (reverse primer); rs4759314, 5’- ACC CAA AAC CAT TTC CTG AGA G -3’ (forward primer) and 5’- TTC AGG TTT TAT TAA CTT GCA TCA GC -3’ (reverse primer). *Hph*I and *Alu*I restriction enzymes were used in this study (Table S1). Taqman probes were obtained directly from Applied Biosystems, and genotyping was performed according to the manufacturer’s protocols. 

### 2.3. Statistical Analysis 

To estimate the relative CAD risk of the various genotypes, the odds ratio (OR) and 95% CI were calculated. The statistical significance of differences between the CAD and control groups was determined by the Student’s *t*-test for continuous variables and the χ2 test for categorical variables. For multivariate analyses, logistic regression analysis was performed to adjust for possible confounders, including age, gender, hypertension, DM, hyperlipidemia, and smoking. A *p*-value less than 0.05 was considered to be a statistically significant difference. Multiple hypothesis testing was performed using the Benjamini–Hochberg method to control the false discovery rate (FDR) in the logistic regression analysis. Calculating the FDR enabled us to address problems associated with multiple comparisons, and FDR provides a measure of the expected proportion of false positives among data. Analyses were performed using GraphPad Prism 4.0 (GraphPad Software, Inc., San Diego, CA, USA), StatsDirect statistical software Version 2.4.4 (StatsDirect Ltd., Altrincham, UK), and MedCalc (Version 7.4 for Windows; MedCalc, Ostend, Belgium). 

The multifactor dimensionality reduction (MDR) method was described previously [21,22]. Briefly, the MDR is comprised of two steps. First, the best combination of multifactors is selected, and then the genotype combinations are classified into high- and low-risk groups [22]. Interaction analyses were performed using the open-source MDR software package (v.2.0) available from www.epistasis.org. (released in 2008) Using these MDR analyses, all possible allelic combinations were identified for gene–gene interactions. HAPSTAT software was used to estimate the frequencies of allelic combinations for the polymorphisms selected by the MDR analysis with strong synergistic effects. HAPSTAT enables testing of haplotype (or allelic combination) effects by maximizing the likelihood (from the observed data) of properly accounting for phase uncertainty and study design. Current versions of the HAPSTAT software (v.3.0) are available from www.bios.unc.edu/~lin/hapstat/ (University of North Carolina, Chapel Hill, NC, USA; released in 2008).

## 3. Results

### 3.1. Characteristics of the Study Population

The baseline characteristics of the CAD and control groups are summarized in Table 1. No significant differences in age and gender distribution were observed between the CAD and control groups, indicating that our frequency matching on age and gender was satisfactory. The average body mass index (BMI) of CAD patients was significantly higher than that of the control group (*p* = 0.0008). In addition, CAD patients exhibited significantly lower serum total cholesterol (*p* < 0.001) and high-density lipoprotein (HDL) cholesterol (*p* = 0.001) levels compared with the control group. However, no difference was observed in the level of serum LDL cholesterol (*p* = 0.896) and triglyceride (TG) (*p* = 0.871) between the two groups. For disease history, 114 (26.1%) patients had a history of DM, which was significantly higher compared with controls (*p* < 0.0001). A significant difference was also observed in the hypertension history between CAD patients and controls (*p* = 0.0007). Nevertheless, no significant differences were found with smoking (*p* = 0.539) or hyperlipidemia history (*p* = 0.263) between CAD patients and the control group.

### 3.2. rs4759314 A>G, rs1899663 G>T, and rs12826786 C>T Polymorphisms Are Significantly Correlated with CAD

In this study, we investigated the distribution of the *HOTAIR* rs4759314 A>G, rs1899663 G>T, rs920778 T>C, rs7958904 G>C, rs12826786 C>T, and rs874945 C>T polymorphisms in CAD patients and the control group (Table 2). The adjusted odds ratio (AOR) from logistic regression analyses with respect to age, gender, hypertension, and DM was calculated. The frequency of each genotype in the control group was consistent with the Hardy–Weinberg equilibrium. Our analysis revealed that several polymorphisms differed significantly between the CAD and control groups, including the rs4759314 A>G (AA vs. AG: crude odds ratio COR, 0.642, 95% CI, 0.414–0.998; AOR, 0.620, 95% CI, 0.395–0.973), rs1899663 G>T (GG vs. TT: COR, 2.276, 95% CI, 1.125–4.607; GG+GT vs. TT: COR, 2.130, 95% CI, 1.046–4.340), and rs12826786 C>T polymorphisms (CC vs. CT: COR, 1.355, 95% CI, 1.017–1.805, AOR, 1.347, 95% CI, 1.005–1.805). However, the rs920778 T>C, rs7958904 G>C, and rs874945 C>T polymorphisms were not significantly different between the CAD and control groups (Table 2).

### 3.3. Relationship between Metabolic Syndrome and Genotype Frequencies

We also conducted a logistic regression analysis to investigate the relationship between metabolic syndrome and the genotype frequency of each polymorphism, with each polymorphism adjusted by age, gender, hypertension, and DM. A comparison between the non-metabolic syndrome control subjects and CAD patients revealed that the rs1899663 G>T and rs12826786 C>T polymorphisms were related to CAD occurrence (rs1899663, GG vs. GT, AOR, 1.577, 95% CI, 1.054–2.361; CC vs. CT: AOR, 1.577, 95% CI, 1.054–2.361). Only the rs1899663 G>T genotype was found to be associated with CAD (rs1899663, GG vs. TT, AOR, 3.522, 95% CI, 1.162–10.676) when non-metabolic syndrome controls and metabolic syndrome patients were compared. However, rs12826786C>T was not significantly associated with CAD prevalence in non-metabolic syndrome control subjects or metabolic syndrome patients (Table 3).

### 3.4. Haplotype Analysis of Polymorphisms with Synergistic Effects

To investigate the genes in the absence of environmental influence, the combined effects of *HOTAIR* alleles and prevalence of CAD were analyzed (Table 4). Our analysis indicated that there were significant effects resulting from combined genes (rs1899663 G>T/rs12826786 C>T) when environmental influence was excluded from CAD risk in the analysis of two SNPs (T-T: OR, 1.448, 95% CI, 1.128–1.859). In our analysis of three SNPs, rs1899663 G>T/rs12826786 C>T/rs874945 C>T and rs1899663 G>T/rs920778 T>C/rs12826786 C>T were significantly different between the CAD and control groups (T-T-C: OR, 1.517, 95% CI, 1.176–1.956; T-C-T: OR, 1.493, 95% CI, 1.151–1.936). The haplotype A-T (rs4759314 A>G rs1899663 G>T) was also found to be significantly associated with CAD risk (A-T: OR, 1.289, 95% CI, 1.021–1.628). When haplotypes, such as A-T-T (rs4759314 A>G/rs1899663 G>T/rs12826786 C>T; *p* = 0.008), A-T-T-T (rs4759314 A>G/rs1899663 G>T/rs12826786 C>T/rs874945 C>T; *p* = 0.0003), and A-T-C-T-T (rs4759314 A>G/rs1899663 G>T/rs920778 T>C/rs12826786 C>T/rs874945 C>T; *p* < 0.0001), were combined, a significant difference between CAD patients and healthy controls was observed (A-T-T: OR, 1.405, 95% CI, 1.092–1.807; A-T-T-T: OR, 1.474, 95% CI, 1.141–1.904; A-T-C-T-T: OR, 1.815, 95% CI, 1.396–2.359).

### 3.5. Interaction Analysis between Genes and Clinical Factors

**Figure 1:**
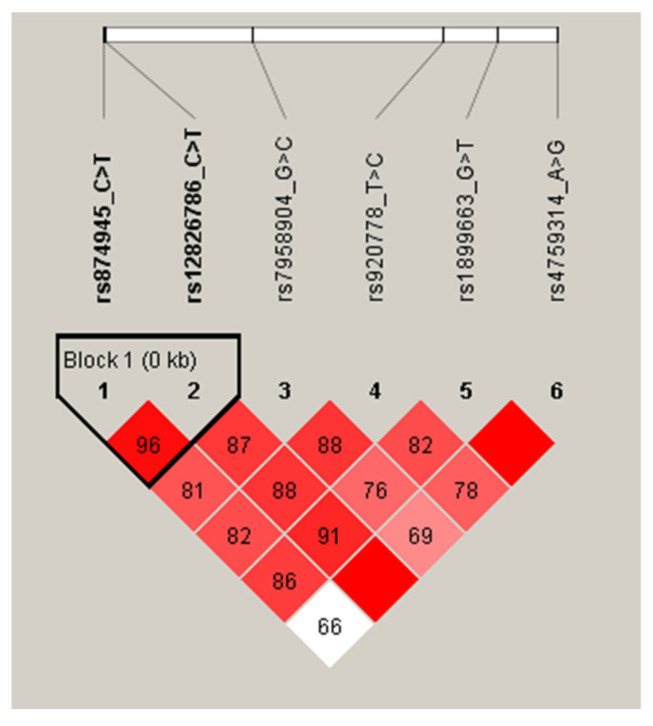
LD patterns of HOTAIR gene polymorphisms in all participants.

We analyzed the possibility of synergistic effects between genes and clinical factors such as metabolic syndrome, DM, hypertension, smoking, folate, and homocysteine (Table 5). Our results showed that rs1899663 has a synergistic effect between DM and metabolic syndrome (rs1899663 GT+TT/DM, AOR = 3.276, 95% CI = 1.786−6.009; rs1899663 GT+TT/metabolic syndrome, AOR = 5.389, 95% CI = 3.272−8.877). When *HOTAIR* rs12826786 CC + TT was combined with hypertension (AOR = 2.012, 95% CI = 1.325−3.057), DM (AOR = 2.755, 95% CI = 1.527−4.968), or metabolic syndrome (AOR = 5.364, 95% CI = 3.244−8.870), we observed an increased association with CAD. Combining the rs920778 TC+CC type with hypertension (AOR = 1.895, 95% CI = 1.274−2.818), DM (AOR = 2.757, 95% CI = 1.604−4.738), and metabolic syndrome (AOR = 5.811, 95% CI = 3.496−9.660) resulted in an increased prevalence towards CAD risk. Figure 1 shows that rs1899663 had the highest synergistic effect with hypertension, metabolic syndrome, and DM, while rs12826786 had the greatest synergy with DM, metabolic syndrome, hypertension, and folate.

### 3.6. Clinical Variables in Ischemic Stroke Patients Stratified by HOTAIR Polymorphism Status 

We conducted a variance analysis between the genes and clinical factors in all participants and subgroups (Table 6). In all participants, the HDL cholesterol level was found to be significantly associated with *HOTAIR* polymorphisms, including rs920778 T>C, rs12826786 C>T, and rs874945 C>T (*p* = 0.040, 0.049, and 0.031, respectively). The TG level demonstrated a decreasing trend depending on the rs1899663 G>T polymorphism (*p* = 0.31). In addition, rs12826786 C>T and rs874945 C>T exhibited a decreasing trend depending on the polymorphisms in the control group (Table 7). In contrast, rs4759314 A>G showed an increasing trend depending on the polymorphisms in the control group. In the CAD group, the TG level was significantly associated with rs920778 T>C (*p* = 0.037), while the HDL cholesterol level was associated with rs12826786 C>T (*p* = 0.023) and rs874945 C>T (*p* = 0.029) (Table 8). Finally, our data demonstrated that rs920778 T>C and rs7958904 G>C displayed a decreasing trend depending on the polymorphisms.

## 4. Discussion

In this study, we investigated the association between CAD occurrence and the lncRNA *HOTAIR*. Our results show that the allelic frequencies of the rs1899663 and rs7958904 SNPs were significantly different between the control and CAD groups in genotype frequencies. These variants have previously been reported in association with other diseases, including various cancers, sclerosis, and psychiatric conditions [7,19,23,24]. Amongst metabolic syndrome patients, rs1899663 and rs12826786 were found to be associated with CAD risk. In our haplotype analysis, rs1899663 G>T and rs12826786 C>T (T-T) were significantly associated with CAD risk. Most of the other haplotypes were significantly associated with protective responses in CAD. These results demonstrate that HOTAIR plays a role in reducing the occurrence of CAD. Further studies are needed to investigate the underlying mechanism of how HOTAIR regulates CAD. 

*HOTAIR*, which is approximately 2.2 kb and consists of six exons, is located between *HOXC11* and *HOXC12* on chromosome 12. Studies have shown that the 5′-end of *HOTAIR* binds PRC2 and the 3′-end binds LSD1, leading to epigenetic changes and transcriptional regulation [9]. At first, *HOTAIR* was reported as a cancer-related gene [6,23,25]; however, further investigation revealed that *HOTAIR* is associated with other conditions as well, including pregnancy, psoriasis, and CAD [5,26,27]. *HOTAIR* overexpression leads to decreased interleukin (IL)-17, IL-23, and tumor necrosis factor alpha, as well as inhibition of nuclear factor kappa B activation in lipopolysaccharide-treated chondrocytes [28]. 

CAD has been associated with endothelial dysfunction, in that endothelial lncRNA influences heart disease prevalence [12,29,30]. *HIF1A-AS2* and *APO1* expression levels were found to be significantly increased in CAD patients [29]. HOTAIR was revealed to be associated with various heart diseases in vivo. It was reported that overexpressed HOTAIR also affects the receptor of advanced glycation end-products (RAGE) that promoted inflammatory responses in an AMI modeling rat [31]. Additionally, HOTAIR was found to regulate oxidative stress, which could progress ischemia-reperfusion (I/R) injury in myocardial model mice [32]. *HOTAIR* is also significantly different between CAD and non-CAD patients. As mentioned, lncRNA functionally associates with microRNAs [12], preventing them from binding target genes. *HOTAIR* inactivates miR-1, which is involved in acute myocardial infarction (AMI) and sponging miR-613 to regulate Connexin 43 in atrial fibrillation, suggesting a protective role for *HOTAIR* in this disease [11,33].

The coronary artery is a blood vessel that supplies oxygen to the heart muscle. CAD results from the blockage of blood flow, which ultimately leads to a lack of oxygen and nutrients to the heart. CAD is caused by several environmental factors, including hyperlipidemia, alcohol, smoking, hypertension, and genetic risk factors [34]. The heritability of CAD risk factors has been reported to increase the occurrence of CAD [35]. Genes reported to be associated with CAD risk are usually related to environmental factors, such as the LDL receptor, apolipoprotein B, and protein convertase subtilisin/kexin type 9. A few reports have suggested that *HOTAIR* is associated with blood vessels or CAD. For example, *HOTAIR* expression was shown to be significantly increased in cardiomyocytes in the septic mouse model [36]. Another study reported that *HOTAIR* was significantly downregulated in the early phase of acute myocardial infarction [33].

Dysfunction of lncRNA is associated with disrupted gene expression and chromatin inactivation [34,35]. Recently, lncRNAs have been proposed as potential biomarkers. The lncRNA autophagy-promoting factor (APF) regulates *ATG7* via miR-188-3p, leading to the regulation of MI [37]. Previous reports demonstrated that *HOTAIR* overexpression is associated with miR-19, suggesting its role as a potential marker in hypertrophy [36]. Moreover, in vivo studies revealed that miR-519d-3p is associated with MI because of the sponging effect of *HOTAIR* [16]. Combining *HOTAIR* with other lncRNAs that have been associated with CAD, such as *APO1*, *HIF1A-AS2*, and *GAS5*, may constitute a novel diagnostic and therapeutic strategy for treating CAD [29,38].This study has several limitations. First, our sample sizes were small and must be verified in a future study utilizing a larger sample population. However, our results were statistically significant. Second, our study population was limited to Korean individuals. Additional investigations including other ethnic groups may reveal different patterns; however, the genotypes of each polymorphism examined must be confirmed to be in HWE. Third, our study was based only on blood samples. The genotypes and allelic frequencies must be confirmed in tissue samples from the placenta or endometrium to exclude the possibility of genetic mosaicism. The effects of *HOTAIR* SNPs should also be confirmed using in vitro studies. Fourth, we analyzed CAD patients and a control group without any history of CAD; however we did not analyze all risk factors such as coagulation factors. For these limitations, we conducted statistical analyses and adjusted for risk factors, including age, gender, hyperlipidemia, diabetes, and hypertension, which affect CAD prevalence, to reduce differences between CAD patients and the control group. In our study population, although there was no significant difference, the LDL level of patients was lower than control group and folate levels were significantly higher in the patients group. As these are unordinary results, they could not exclude that patients may have an additional regimen or nutritional supplement to improve folate and LDL levels.

## 5. Conclusions

We demonstrated that the *HOTAIR* rs4759314 A>G, rs1899663 C>T, and rs12826786 C>T polymorphisms are genetically associated with CAD in the Korean population. The rs1899663 and rs12826786 polymorphisms were associated with increased CAD risk, but rs4759314 was linked to a decreased CAD risk. This study suggests that the *HOTAIR* polymorphisms may contribute to CAD and may be potential biomarkers for assessing CAD risk. Further studies using larger cohorts are required to better understand the role of *HOTAIR* as a biomarker. Moreover, in vivo and vitro studies are necessary to learn which function of HOTAIR influences downstream gene expression and ultimately leads to CAD. This knowledge would help in CAD diagnosis and could ultimately lead to the development of effective therapeutic strategies.

## Figures and Tables

**Table 1:** Baseline characteristics of control subjects and coronary artery disease patients.

Characteristics	Controls (*n* = 418)	CAD (*n* = 442)	*P^a^*
Age (years, mean ± SD)	61.43 ± 11.15	61.35 ± 11.50	0.521
Male (%)	172 (41.1)	186 (42.1)	0.084
Hypertension (%)	169 (40.4)	228 (52.2)	0.0007
Systolic BP (mmHg, mean ± SD)	131.47 ± 17.21	127.75 ± 20.58	<0.001
Diastolic BP (mmHg, mean ± SD)	80.33 ± 11.84	78.77 ± 12.81	0.101
Diabetes mellitus (%)	59 (14.1)	114 (26.1)	<0.0001
Fasting blood sugar (mg/dL, mean ± SD)	113.93 ± 37.51	141.02 ± 62.84	<0.001
Hemoglobin A1c (%, mean ± SD)	6.24 ± 1.44	6.42 ± 1.51	0.568
Hyperlipidemia (*n*, %)	100 (23.9)	121 (27.5)	0.263
BMI ≥ 25 kg/m^2^, *n* (%)	103 (38.3)	224 (51.6)	0.0008
Total cholesterol (mg/dL, mean ± SD)	192.15 ± 37.23	187.37 ± 47.04	<0.001
Triglyceride (mg/dL, mean ± SD)	146.92 ± 91.26	158.21 ± 90.55	0.871
LDL cholesterol (mg/dL, mean ± SD)	116.16 ± 41.36	112.49 ± 41.07	0.896
HDL cholesterol (mg/dL, mean ± SD)	46.37 ± 13.77	43.68 ± 11.21	0.001
Smokers (%)	138 (33.0)	135 (30.8)	0.539
Folate (nmol/L, mean ± SD)	8.77 ± 6.26	8.78 ± 9.76	<0.001
Vitamin B12 (pg/mL, mean ± SD)	686.11 ± 268.65	693.47 ± 306.38	0.102
Homocysteine (μmol/L, mean ± SD)	9.84 ± 4.14	9.65 ± 4.67	0.014

Note: CAD, coronary artery disease; SD, standard deviation; HDL, high-density lipoprotein; LDL, low-density lipoprotein. *P^a^* was calculated using the Mann–Whitney test for continuous variables and Chi-square test for categorical variables.

**Table 2:** Comparison of genotype frequencies and AOR values of *HOTAIR* polymorphisms between the CAD and control subjects.

Genotypes	Controls (*n* = 418)	CAD (*n* = 442)	COR (95% CI)	*p*	AOR (95% CI)	*p*
rs4759314 A>G						
AA	362 (86.6)	404 (76.2)	1.000 (reference)			
AG	53 (12.7)	38 (7.2)	0.642 (0.414–0.998)	0.049	0.620 (0.395–0.973)	0.037
GG	3 (0.7)	0 (0.0)	N/A	N/A	N/A	N/A
Dominant (AA vs. AG+GG)			0.608 (0.393–0.940)	0.025	0.586 (0.375–0.916)	0.019
Recessive (AA+AG vs. GG)			N/A	N/A	N/A	N/A
HWE-P	0.494	0.345				
rs1899663 G>T						
GG	270 (64.6)	257 (48.5)	1.000 (reference)			
GT	136 (32.5)	159 (30.0)	1.228 (0.923–1.634)	0.158	1.245 (0.929–1.667)	0.142
TT	12 (2.9)	26 (4.9)	2.276 (1.125–4.607)	0.022	2.284 (1.110–4.700)	0.025
Dominant (GG vs. GT+TT)			1.313 (0.997–1.730)	0.053	1.336 (1.008–1.772)	0.044
Recessive (GG+GT vs. TT)			2.115 (1.053–4.248)	0.035	2.140 (1.052–4.357)	0.036
HWE-P	0.295	0.830				
rs920778 T>C						
TT	243 (58.1)	231 (43.6)	1.000 (reference)			
TC	151 (36.1)	184 (34.7)	1.282 (0.968–1.697)	0.083	1.261 (0.946–1.680)	0.114
CC	24 (5.7)	27 (5.1)	1.183 (0.664–2.111)	0.568	1.167 (0.646–2.110)	0.608
Dominant (TT vs. TC+CC)			1.268 (0.969–1.661)	0.084	1.251 (0.950–1.647)	0.111
Recessive (TT+TC vs. CC)			1.068 (0.606–1.883)	0.820	1.062 (0.596–1.892)	0.839
HWE-P	0.932	0.223				
rs7958904 G>C						
GG	250 (59.8)	246 (46.4)	1.000 (reference)			
GC	139 (33.3)	167 (31.5)	1.221 (0.918–1.625)	0.171	1.187 (0.886–1.591)	0.25
CC	29 (6.9)	29 (5.5)	1.016 (0.590–1.751)	0.954	0.980 (0.561–1.709)	0.942
Dominant (GG vs. GC+CC)			1.186 (0.904–1.555)	0.218	1.153 (0.874–1.520)	0.315
Recessive (GG+GC vs. CC)			0.942 (0.553–1.605)	0.826	0.917 (0.533–1.577)	0.753
HWE-P	0.116	0.927				
rs12826786 C>T						
CC	282 (67.5)	266 (50.2)	1.000 (reference)			
CT	126 (30.1)	161 (30.4)	1.355 (1.017–1.805)	0.038	1.338 (0.998–1.794)	0.052
TT	10 (2.4)	15 (2.8)	1.590 (0.702–3.602)	0.266	1.589 (0.686–3.679)	0.28
Dominant (CC vs. CT+TT)			1.372 (1.037–1.814)	0.027	1.360 (1.022–1.810)	0.035
Recessive (CC+CT vs. TT)			1.433 (0.637–3.227)	0.385	1.472 (0.643–3.369)	0.36
HWE-P	0.351	0.114				
rs874945 C>T						
CC	267 (63.9)	258 (48.7)	1.000 (reference)			
CT	133 (31.8)	164 (30.9)	1.276 (0.959–1.698)	0.094	1.249 (0.932–1.673)	0.137
TT	18 (4.3)	20 (3.8)	1.150 (0.595–2.223)	0.678	1.131 (0.573–2.230)	0.723
Dominant (CC vs. CT+TT)			1.261 (0.958–1.660)	0.098	1.246 (0.940–1.651)	0.126
Recessive (CC+CT vs. TT)			1.053 (0.549–2.020)	0.876	1.078 (0.555–2.095)	0.824
HWE-P	0.781	0.343				

Note: AOR was adjusted by age, gender, hypertension, diabetes mellitus, and hyperlipidemia. COR = crude odds ratio; AOR = adjusted odds ratio; 95% CI = 95% confidence interval; HWE = Hardy–Weinberg equilibrium.

**Table 3:** Comparison of genotype frequencies and AOR values of *HOTAIR* polymorphisms between metabolic syndromes.

Genotypes	Non-MetS Controls (*n* = 288)	Non-MetS CAD (*n* = 172)	AOR (95% CI)	*p*	MetS Controls (*n* = 121)	MetS CAD (*n* = 267)	AOR (95% CI)	*p*	Non-MetS Controls (*n* = 288)	MetS CAD (*n* = 267)	AOR (95% CI)	*p*
rs4759314 A>G												
AA	252 (87.5)	160 (93.0)	1.000 (reference)		103 (85.1)	241 (90.3)	1.000 (reference)		252 (87.5)	241 (90.3)	1.000 (reference)	
AG	34 (11.8)	12 (7.0)	0.562 (0.281–1.124)	0.103	17 (14.0)	26 (9.7)	0.699 (0.358–1.368)	0.296	34 (11.8)	26 (9.7)	0.746 (0.401–1.387)	0.354
GG	2 (0.7)	0 (0.0)	N/A	N/A	1 (0.8)	0 (0.0)	N/A	N/A	2 (0.7)	0 (0.0)	N/A	N/A
Dominant (AA vs. AG+GG)			0.535 (0.269–1.065)	0.075			0.657 (0.340–1.273)	0.213			0.700 (0.380–1.292)	0.254
Recessive (AA+AG vs. GG)			N/A	N/A			N/A	N/A			N/A	N/A
HWE-P												
rs1899663 G>T												
GG	191 (66.3)	94 (54.7)	1.000 (reference)		77 (63.6)	161 (60.3)	1.000 (reference)		191 (66.3)	161 (60.3)	1.000 (reference)	
GT	91 (31.6)	70 (40.7)	1.577 (1.054–2.361)	0.027	38 (31.4)	88 (33.0)	1.135 (0.703–1.831)	0.604	91 (31.6)	88 (33.0)	1.238 (0.820–1.870)	0.310
TT	6 (2.1)	8 (4.7)	2.400 (0.787–7.318)	0.124	6 (5.0)	18 (6.7)	1.678 (0.616–4.575)	0.312	6 (2.1)	18 (6.7)	3.522 (1.162–10.676)	0.026
Dominant (GG vs. GT+TT)			1.642 (1.108–2.432)	0.013			1.221 (0.774–1.926)	0.391			1.381 (0.927–2.057)	0.113
Recessive (GG+GT vs. TT)			2.074 (0.692–6.218)	0.193			1.630 (0.614–4.323)	0.327			3.178 (1.095–9.218)	0.033
HWE-P												
rs920778 T>C												
TT	167 (58.0)	88 (51.2)	1.000 (reference)		73 (60.3)	142 (53.2)	1.000 (reference)		167 (58.0)	142 (53.2)	1.000 (reference)	
TC	108 (37.5)	73 (42.4)	1.300 (0.870–1.942)	0.201	37 (30.6)	109 (40.8)	1.587 (0.986–2.555)	0.057	108 (37.5)	109 (40.8)	1.113 (0.747–1.659)	0.598
CC	13 (4.5)	11 (6.4)	1.573 (0.665–3.720)	0.303	11 (9.1)	16 (6.0)	0.800 (0.344–1.858)	0.604	13 (4.5)	16 (6.0)	1.567 (0.629–3.901)	0.335
Dominant (TT vs. TC+CC)			1.340 (0.910–1.974)	0.139			1.424 (0.911–2.225)	0.121			1.157 (0.788–1.699)	0.456
Recessive (TT+TC vs. CC)			1.417 (0.614–3.270)	0.414			0.701 (0.310–1.587)	0.394			1.468 (0.619–3.483)	0.384
HWE-P												
rs7958904 G>C												
GG	173 (60.1)	89 (51.7)	1.000 (reference)		74 (61.2)	156 (58.4)	1.000 (reference)		173 (60.1)	156 (58.4)	1.000 (reference)	
GC	97 (33.7)	73 (42.4)	1.449 (0.967–2.170)	0.072	36 (29.8)	92 (34.5)	1.246 (0.769–2.020)	0.372	97 (33.7)	92 (34.5)	0.975 (0.646–1.472)	0.904
CC	18 (6.3)	10 (5.8)	0.986 (0.423–2.296)	0.974	11 (9.1)	19 (7.1)	0.864 (0.384–1.947)	0.725	18 (6.3)	19 (7.1)	1.040 (0.469–2.304)	0.924
Dominant (GG vs. GC+CC)			1.395 (0.946–2.056)	0.093			1.164 (0.743–1.822)	0.508			0.994 (0.674–1.466)	0.975
Recessive (GG+GC vs. CC)			0.870 (0.387–1.956)	0.735			0.814 (0.368–1.799)	0.611			1.093 (0.508–2.350)	0.820
HWE-P												
rs12826786 C>T												
CC	197 (68.4)	99 (57.6)	1.000 (reference)		81 (66.9)	166 (62.2)	1.000 (reference)		197 (68.4)	166 (62.2)	1.000 (reference)	
CT	84 (29.2)	69 (40.1)	1.633 (1.089–2.450)	0.018	37 (30.6)	90 (33.7)	1.211 (0.754–1.946)	0.428	84 (29.2)	90 (33.7)	1.283 (0.847–1.944)	0.240
TT	7 (2.4)	4 (2.3)	1.117 (0.310–4.025)	0.866	3 (2.5)	11 (4.1)	1.965 (0.513–7.526)	0.324	7 (2.4)	11 (4.1)	1.506 (0.483–4.696)	0.480
Dominant (CC vs. CT+TT)			1.597 (1.073–2.377)	0.021			1.275 (0.804–2.022)	0.303			1.311 (0.877–1.960)	0.187
Recessive (CC+CT vs. TT)			0.926 (0.262–3.274)	0.905			1.832 (0.491–6.836)	0.367			1.499 (0.500–4.489)	0.470
HWE-P												
rs874945 C>T												
CC	185 (64.2)	98 (57.0)	1.000 (reference)		78 (64.5)	159 (59.6)	1.000 (reference)		185 (64.2)	159 (59.6)	1.000 (reference)	
CT	91 (31.6)	68 (39.5)	1.392 (0.928–2.087)	0.11	37 (30.6)	94 (35.2)	1.296 (0.805–2.086)	0.286	91 (31.6)	94 (35.2)	1.222 (0.811–1.841)	0.339
TT	12 (4.2)	6 (3.5)	0.752 (0.262–2.155)	0.596	6 (5.0)	14 (5.2)	1.323 (0.474–3.694)	0.593	12 (4.2)	14 (5.2)	1.522 (0.585–3.960)	0.389
Dominant (CC vs. CT+TT)			1.322 (0.893–1.958)	0.164			1.321 (0.837–2.085)	0.233			1.260 (0.848–1.870)	0.253
Recessive (CC+CT vs. TT)			0.713 (0.256–1.988)	0.518			1.233 (0.452–3.364)	0.682			1.451 (0.583–3.611)	0.424
HWE-P												

Note: AOR was adjusted by age, gender, hypertension, and diabetes mellitus. AOR = adjusted odds ratio; 95% CI = 95% confidence interval; HWE = Hardy–Weinberg equilibrium.

**Table 4:** Haplotype analysis of *HOTAIR* polymorphisms in CAD and controls subjects.

Haplotype	Controls (2*n* = 836)	Case (2*n* = 884)	OR (95% CI)	*p*-Value
rs4759314 A>G/ rs1899663 G>T/ rs920778 T>C/ rs7958904 G>C/ rs12826786 C>T/ rs874945 C>T				
A-G-T-G-C-C	0.6855	0.7014	1.000 (reference)	
A-G-T-G-C-T	0.0137	0.0012	0.084 (0.011–0.653)	0.003
A-G-T-C-C-C	0.0109	0.0022	0.205 (0.044–0.955)	0.033
A-G-T-C-T-T	0.0062	0.0000	0.084 (0.005–1.524)	0.026
A-T-C-C-T-C	0.0081	0.0000	0.062 (0.004–1.082)	0.006
A-T-C-C-T-T	0.1197	0.1909	1.562 (1.190–2.050)	0.001
G-G-T-G-C-C	0.0088	0.0000	0.062 (0.004–1.082)	0.006
rs4759314 A>G/ rs1899663 G>T/ rs920778 T>C/ rs7958904 G>C/ rs12826786 C>T				
A-G-T-G-C	0.6992	0.7025	1.000 (reference)	
A-G-T-C-C	0.0121	0.0022	0.188 (0.041–0.864)	0.019
A-G-T-C-T	0.0064	0.0000	0.086 (0.005–1.553)	0.027
A-T-C-C-T	0.1269	0.1909	1.502 (1.149–1.963)	0.003
G-G-T-G-C	0.0098	0.0000	0.055 (0.003–0.963)	0.003
rs4759314 A>G/ rs1899663 G>T/ rs920778 T>C/ rs12826786 C>T/ rs874945 C>T				
A-G-T-C-C	0.6949	0.7034	1.000 (reference)	
A-G-T-C-T	0.0149	0.0012	0.091 (0.012–0.704)	0.004
A-G-T-T-T	0.0095	0.0000	0.064 (0.004–1.119)	0.008
A-T-C-T-C	0.0094	0.0000	0.064 (0.004–1.119)	0.008
A-T-C-T-T	0.1287	0.2023	1.815 (1.396–2.359)	<0.0001
G-G-T-C-C	0.0100	0.0012	0.137 (0.017–1.098)	0.041
rs4759314 A>G/ rs1899663 G>T/ rs7958904 G>C/ rs12826786 C>T/ rs874945 C>T				
A-G-G-C-C	0.6923	0.7045	1.000 (reference)	
A-G-G-C-T	0.0136	0.0025	0.169 (0.037–0.766)	0.011
A-G-C-T-T	0.0160	0.0046	0.286 (0.093–0.882)	0.026
A-T-C-T-C	0.0094	0.0000	0.055 (0.003–0.950)	0.003
A-T-C-T-T	0.1275	0.1955	1.503 (1.151–1.961)	0.003
G-G-G-C-C	0.0122	0.0023	0.186 (0.041–0.852)	0.019
rs4759314 A>G/ rs1899663 G>T/ rs920778 T>C/ rs12826786 C>T				
A-G-T-C	0.7088	0.7047	1.000 (reference)	
A-G-T-T	0.0097	0.0000	0.056 (0.003–0.973)	0.003
A-T-C-T	0.1378	0.2022	1.482 (1.142–1.921)	0.003
G-G-T-C	0.0112	0.0012	0.106 (0.013–0.838)	0.010
rs4759314 A>G/ rs1899663 G>T/ rs7958904 G>C/ rs12826786 C>T				
A-G-G-C	0.7055	0.7068	1.000 (reference)	
A-G-C-T	0.0167	0.0046	0.270 (0.088–0.824)	0.017
A-T-C-T	0.1365	0.1954	1.433 (1.102–1.861)	0.007
G-G-G-C	0.0132	0.0036	0.258 (0.071–0.928)	0.031
rs4759314 A>G/ rs1899663 G>T/ rs12826786 C>T/ rs874945 C>T				
A-G-C-C	0.7050	0.7108	1.000 (reference)	
A-G-C-T	0.0149	0.0024	0.156 (0.035–0.702)	0.006
A-G-T-T	0.0216	0.0046	0.208 (0.070–0.620)	0.002
A-T-T-C	0.0099	0.0000	0.055 (0.003–0.959)	0.003
A-T-T-T	0.1425	0.2114	1.474 (1.141–1.904)	0.003
rs4759314 A>G/ rs1899663 G>T/ rs12826786 C>T				
A-G-C	0.7187	0.7131	1.000 (reference)	
A-G-T	0.0221	0.0047	0.212 (0.071–0.630)	0.002
A-T-T	0.1522	0.2114	1.405 (1.092–1.807)	0.008
G-G-C	0.0675	0.0424	0.630 (0.410–0.969)	0.034
rs1899663 G>T/ rs12826786 C>T				
G-C	0.7861	0.7555	1.000 (reference)	
G-T	0.0225	0.0047	0.207 (0.070–0.612)	0.002
T-T	0.1522	0.2114	1.448 (1.128–1.859)	0.004

CAD, coronary artery disease; OR, odds ratio; CI, confidence interval.

**Table 5:** Interaction analysis between genotype and patients characteristics in participants.

Characteristics	rs1899663 GG	*p*	rs1899663 GG+GT	*p*	rs920778 TT	*p*	rs920778 TT+TC	*p*
Hypertension								
No	1.000 (reference)		1.230 (0.834–1.814)	0.296	1.000 (reference)		1.217 (0.833–1.779)	0.310
Yes	1.424 (0.989–2.049)	0.057	1.967 (1.302–2.972)	0.001	1.514 (1.029–2.227)	0.036	1.895 (1.274–2.818)	0.002
Diabetes mellitus								
No	1.000 (reference)		1.275 (0.934–1.742)	0.126	1.000 (reference)		1.236 (0.911–1.679)	0.174
Yes	1.969 (1.262–3.071)	0.003	3.276 (1.786–6.009)	0.000	2.110 (1.308–3.405)	0.002	2.757 (1.604–4.738)	0.000
Smoking								
No	1.000 (reference)		1.276 (0.902–1.805)	0.169	1.000 (reference)		1.234 (0.880–1.731)	0.224
Yes	0.724 (0.466–1.124)	0.150	1.165 (0.699–1.942)	0.558	0.697 (0.435–1.118)	0.135	1.009 (0.613–1.660)	0.973
Metabolic syndrome								
No	1.000 (reference)		1.533 (1.040–2.262)	0.031	1.000 (reference)		1.283 (0.874–1.882)	0.203
Yes	4.898 (3.169–7.570)	<0.0001	5.389 (3.272–8.877)	<0.0001	3.989 (2.536–6.275)	<0.0001	5.811 (3.496–9.660)	<0.0001
BMI								
<25 kg/m^2^	1.000 (reference)		1.591 (1.108–2.284)	0.012	1.000 (reference)		1.356 (0.951–1.933)	0.092
≥25 kg/m^2^	3.900 (2.646–5.749)	<0.0001	3.479 (2.243–5.394)	<0.0001	3.588 (2.375–5.420)	<0.0001	3.560 (2.331–5.438)	<0.0001
Triglycerides								
<150 mg/dL	1.000 (reference)		0.955 (0.667–1.365)	0.799	1.000 (reference)		0.949 (0.668–1.349)	0.772
≥150 mg/dL	0.949 (0.661–1.364)	0.778	2.223 (1.423–3.473)	0.000	0.927 (0.632–1.361)	0.700	1.844 (1.210–2.810)	0.004
Folate								
>4.01 nmol/L	1.000 (reference)		1.381 (1.016–1.877)	0.039	1.000 (reference)		1.251 (0.928–1.688)	0.142
≤4.01 nmol/L	2.343 (1.323–4.151)	0.004	2.109 (1.165–3.820)	0.014	2.142 (1.153–3.978)	0.016	2.097 (1.198–3.669)	0.010
**Characteristics**	**rs7958904 GG**	***p***	**rs7958904 GG+GC**	***p***	**rs12826786 CC**	***p***	**rs12826786 CC+CT**	***p***
Hypertension								
No	1.000 (reference)		1.135 (0.774–1.665)	0.516	1.000 (reference)		1.376 (0.928–2.041)	0.113
Yes	1.506 (1.031–2.202)	0.034	1.797 (1.201–2.688)	0.004	1.512 (1.057–2.163)	0.024	2.012 (1.325–3.057)	0.001
Diabetes mellitus								
No	1.000 (reference)		1.197 (0.880–1.630)	0.252	1.000 (reference)		1.392 (1.014–1.910)	0.041
Yes	2.307 (1.428–3.727)	0.001	2.300 (1.353–3.910)	0.002	2.188 (1.403–3.410)	0.001	2.755 (1.527–4.968)	0.001
Smoking								
No	1.000 (reference)		1.226 (0.872–1.724)	0.242	1.000 (reference)		1.357 (0.954–1.931)	0.089
Yes	0.801 (0.505–1.270)	0.345	0.913 (0.556–1.499)	0.720	0.750 (0.484–1.164)	0.200	1.108 (0.664–1.848)	0.695
Metabolic syndrome								
No	1.000 (reference)		1.338 (0.911–1.966)	0.138	1.000 (reference)		1.539 (1.038–2.281)	0.032
Yes	4.296 (2.769–6.664)	<0.0001	5.745 (3.399–9.709)	<0.0001	4.464 (2.921–6.823)	<0.0001	5.364 (3.244–8.870)	<0.0001
BMI								
<25 kg/m^2^	1.000 (reference)		1.287 (0.902–1.836)	0.164	1.000 (reference)		1.616 (1.120–2.331)	0.010
≥25 kg/m^2^	3.485 (2.339–5.192)	<0.0001	3.503 (2.258–5.436)	<0.0001	3.779 (2.581–5.535)	<0.0001	3.477 (2.228–5.426)	<0.0001
Triglycerides								
<150 mg/dL	1.000 (reference)		0.962 (0.674–1.373)	0.830	1.000 (reference)		1.026 (0.712–1.478)	0.890
≥150 mg/dL	1.027 (0.703–1.499)	0.892	1.609 (1.063–2.436)	0.025	1.003 (0.700–1.436)	0.989	2.129 (1.368–3.314)	0.001
^†^ Folate								
>4.01 nmol/L	1.000 (reference)		1.184 (0.877–1.600)	0.271	1.000 (reference)		1.369 (1.004–1.868)	0.048
≤4.01 nmol/L	2.268 (1.246–4.129)	0.007	1.897 (1.065–3.379)	0.030	2.056 (1.185–3.567)	0.010	2.360 (1.263–4.408)	0.007

CAD, coronary artery disease; AOR, adjusted odds ratio; 95% CI, 95% confidence interval. The adjusted odds ratio on the basis of risk factors, such as age, gender, hypertension, and diabetes mellitus. † Folate 4.01 nmol/L is lower than the 15% cut-off for each level in CAD patients and controls.

**Table 6:** Clinical variables in CAD patients and controls stratified by *HOTAIR* polymorphisms status by ANOVA.

Genotypes	BMI (kg/m^2^)	Total Cholesterol (mg/dL)	Triglyceride (mg/dL)	HDL Cholsterol (mg/dL)	LDL Cholesterol (mg/dL)	FBS (mg/dL)	SBP (mmHg)	DBP (mmHg)	Folate (mg/mL)	Vitamin B12 (pg/mL)	Homocysteine (mmol/L)
	Mean ± SD (703)	Mean ± SD (846)	Mean ± SD (846)	Mean ± SD (616)	Mean ± SD (602)	Mean ± SD (838)	Mean ± SD (860)	Mean ± SD (860)	Mean ± SD (817)	Mean ± SD (502)	Mean ± SD (836)
rs4759314 A>G											
AA	24.82 ± 3.41	189.65 ± 43.00	152.63 ± 92.05	44.64 ± 12.03	113.60 ± 41.47	127.39 ± 53.30	129.91 ± 18.97	79.85 ± 12.27	8.77 ± 8.38	689.81 ± 269.93	9.71 ± 4.35
AG	24.70 ± 3.11	188.89 ± 38.66	152.11 ± 82.23	42.99 ± 12.36	113.28 ± 38.67	132.68 ± 58.30	126.62 ± 20.15	76.81 ± 12.87	8.80 ± 6.28	673.49 ± 317.61	10.02 ± 4.98
GG	24.93 ± 3.83	218.33 ± 74.00	208.67 ± 86.23			95.33 ± 2.08	128.33 ± 16.07	78.33 ± 15.28	9.29 ± 5.27	613.00 ± 191.27	10.45 ± 3.98
*p*-value	0.953	0.501	0.567	0.291	0.952	0.395	0.296	0.084	0.993	0.818	0.789
rs1899663 G>T											
GG	24.65 ± 3.39	189.04 ± 40.11	151.70 ± 90.66	43.85 ± 11.33	113.91 ± 40.65	128.36 ± 54.41	130.00 ± 19.50	79.59 ± 12.65	8.69 ± 6.82	682.64 ± 258.63	9.68 ± 4.36
GT	25.07 ± 3.41	192.02 ± 45.02	154.67 ± 91.12	45.46 ± 13.60	113.10 ± 40.52	124.10 ± 50.44	129.19 ± 18.22	79.54 ± 11.96	9.20 ± 10.42	689.76 ± 300.19	9.90 ± 4.55
TT	25.01 ± 3.00	180.58 ± 55.82	153.24 ± 97.16	44.77 ± 9.07	112.89 ± 50.21	149.67 ± 65.10	126.32 ± 20.22	78.50 ± 11.70	6.55 ± 4.18	768.43 ± 349.37	9.35 ± 4.18
*p*-value	0.296	0.066	0.906	0.297	0.970	0.019	0.476	0.871	0.190	0.518	0.681
rs920778 T>C											
TT	24.66 ± 3.33	189.08 ± 40.95	151.48 ± 90.00	43.37 ± 10.75	114.91 ± 41.76	127.62 ± 53.97	130.12 ± 19.38	79.78 ± 12.48	8.69 ± 6.79	609.68 ± 254.33	9.56 ± 4.21
TC	24.98 ± 3.54	191.49 ± 45.31	155.89 ± 93.60	45.94 ± 13.55	113.46 ± 41.99	127.53 ± 53.50	129.22 ± 18.67	79.60 ± 12.42	9.09 ± 10.16	685.32 ± 280.53	9.94 ± 4.57
CC	25.06 ± 2.54	183.35 ± 40.27	144.69 ± 84.10	44.36 ± 11.93	103.09 ± 28.13	131.78 ± 54.62	126.57 ± 19.17	76.69 ± 10.60	7.56 ± 4.96	696.29 ± 273.63	10.15 ± 5.24
*p*-value	0.576	0.406	0.644	0.040	0.238	0.866	0.413	0.235	0.448	0.281	0.399
rs7958904 G>C											
GG	24.73 ± 3.38	188.83 ± 41.54	150.37 ± 88.44	43.36 ± 10.76	115.25 ± 41.65	127.35 ± 53.73	129.79 ± 19.61	79.41 ± 12.60	8.80 ± 6.83	669.91 ± 275.67	9.52 ± 4.09
GC	24.83 ± 3.40	192.15 ± 45.05	157.42 ± 95.09	46.03 ± 13.82	112.32 ± 42.21	127.73 ± 54.41	128.91 ± 17.38	79.85 ± 11.84	8.98 ± 10.39	673.32 ± 274.47	10.04 ± 4.70
CC	25.37 ± 3.26	184.00 ± 38.90	149.17 ± 91.64	45.00 ± 11.43	107.17 ± 30.85	132.47 ± 51.46	130.95 ± 23.12	78.81 ± 13.24	7.53 ± 4.85	697.38 ± 276.20	10.04 ± 5.40
*p*-value	0.466	0.330	0.546	0.095	0.397	0.793	0.693	0.802	0.477	0.621	0.327
rs12826786 C>T											
CC	24.65 ± 3.41	188.63 ± 40.16	150.74 ± 90.27	43.56 ± 11.15	113.88 ± 40.22	128.08 ± 54.68	129.71 ± 19.63	79.33 ± 12.52	8.69 ± 6.75	683.65 ± 267.04	9.68 ± 4.29
CT	25.05 ± 3.36	193.24 ± 47.50	157.98 ± 94.52	46.10 ± 13.66	114.77 ± 44.08	127.00 ± 52.48	129.30 ± 18.30	80.05 ± 12.26	9.16 ± 10.58	698.35 ± 292.93	9.88 ± 4.63
TT	25.52 ± 2.58	172.04 ± 31.84	138.28 ± 61.58	44.39 ± 9.56	97.18 ± 22.10	132.00 ± 50.08	129.20 ± 16.63	77.84 ± 9.96	6.30 ± 4.06	643.20 ± 286.57	9.60 ± 4.68
*p*-value	0.205	0.069	0.403	0.049	0.166	0.895	0.954	0.576	0.230	0.756	0.822
rs874945 C>T											
CC	24.68 ± 3.43	188.97 ± 39.82	150.22 ± 90.50	43.49 ± 11.23	114.64 ± 40.21	128.35 ± 54.99	129.91 ± 19.75	79.56 ± 12.58	8.58 ± 6.70	683.42 ± 267.15	9.71 ± 4.46
CT	25.01 ± 3.37	193.02 ± 47.76	158.11 ± 93.62	46.19 ± 13.43	113.63 ± 44.35	125.22 ± 50.56	129.07 ± 18.22	79.74 ± 12.31	9.37 ± 10.58	693.50 ± 286.89	9.84 ± 4.39
TT	25.02 ± 2.73	173.66 ± 34.78	146.95 ± 77.00	44.01 ± 10.51	100.30 ± 24.21	141.03 ± 60.20	128.42 ± 16.73	77.45 ± 9.52	6.86 ± 4.52	702.25 ± 320.37	9.51 ± 4.14
*p*-value	0.437	0.040	0.459	0.031	0.188	0.228	0.776	0.559	0.064	0.905	0.875

ANOVA, analysis of variance; HDL cholesterol, high-density lipoprotein cholesterol; LDL cholesterol, low-density lipoprotein cholesterol; BMI, body mass index; SD, standard deviation.

**Table 7:** Clinical variables in healthy controls stratified by *HOTAIR* polymorphisms status by ANOVA.

Genotypes	BMI (kg/m^2^)	Total Cholesterol (mg/dL)	Triglyceride (mg/dL)	HDL Cholesterol (mg/dL)	LDL Cholesterol (mg/dL)	FBS (mg/dL)	SBP (mmHg)	DBP (mmHg)	Folate (mg/mL)	Vitamin B12 (pg/mL)	Homocysteine (mmol/L)
	Mean ± SD (703)	Mean ± SD (846)	Mean ± SD (846)	Mean ± SD (616)	Mean ± SD (602)	Mean ± SD (838)	Mean ± SD (860)	Mean ± SD (860)	Mean ± SD (817)	Mean ± SD (502)	Mean ± SD (836)
rs4759314 A>G											
AA	24.35 ± 3.46	191.43 ± 36.44	146.27 ± 92.22	47.02 ± 13.75	114.34 ± 41.33	112.37 ± 33.27	132.35 ± 16.99	80.74 ± 11.67	8.81 ± 6.39	685.91± 261.73	9.85 ± 4.14
AG	24.38 ± 3.16	195.59 ± 40.39	147.77 ± 85.06	43.00 ± 13.61	125.45 ± 41.00	125.86 ± 58.78	125.66 ± 17.89	77.66 ± 12.65	8.41 ± 5.41	691.77± 319.34	9.73 ± 4.24
GG	24.93 ± 3.83	218.33 ± 74.00	208.67 ± 86.23	N/A	N/A	95.33 ± 2.08	128.33 ± 16.07	78.33 ± 15.28	9.29 ± 5.27	613.00± 191.27	10.45 ± 3.98
*p*-value	0.956	0.36	0.499	0.151	0.187	0.507	0.029	0.202	0.9	0.885	0.951
rs1899663 G>T											
GG	24.01 ± 3.25	192.45 ± 37.48	151.97 ± 93.64	45.74 ± 11.89	117.24 ± 47.04	114.64 ± 40.35	131.75 ± 17.87	80.29 ± 12.29	8.55 ± 5.77	675.01± 246.34	9.85 ± 4.23
GT	25.04 ± 3.61	193.26 ± 37.62	137.75 ± 85.43	47.81 ± 17.88	115.24 ± 30.78	111.36 ± 31.87	130.15 ± 15.86	79.82 ± 11.03	9.37 ± 7.24	701.31± 302.31	9.78 ± 3.91
TT	24.91 ± 3.91	173.50 ± 21.31	134.50 ± 97.95	45.64 ± 5.46	109.00 ± 16.43	125.83 ± 24.99	140.00 ± 14.92	86.92 ± 8.39	6.87 ± 4.09	757.75± 336.43	10.31 ± 4.92
*p*-value	0.067	0.208	0.31	0.65	0.819	0.385	0.148	0.137	0.266	0.423	0.914
rs920778 T>C											
TT	24.13 ± 3.10	191.22 ± 36.79	152.94 ± 96.93	44.67 ± 10.61	116.29 ± 46.15	112.51 ± 35.35	132.49 ± 17.31	80.47 ± 11.90	8.62 ± 5.79	610.21± 228.97	9.70 ± 3.95
TC	24.63 ± 3.88	193.36 ± 38.10	136.85 ± 79.69	48.84 ± 17.84	117.17 ± 36.09	116.45 ± 42.65	129.24 ± 17.12	80.22 ± 12.16	9.09 ± 7.11	696.09± 278.25	10.00 ± 4.47
CC	24.85 ± 2.85	194.04 ± 37.57	148.67 ± 97.77	47.50 ± 11.73	110.23 ± 25.34	112.96 ± 22.71	135.21 ± 15.78	79.63 ± 9.25	8.23 ± 5.15	687.57± 266.04	10.27 ± 4.06
*p*-value	0.447	0.833	0.437	0.158	0.859	0.603	0.104	0.937	0.709	0.346	0.685
rs7958904 G>C											
GG	24.12 ± 3.25	191.93 ± 37.48	149.51 ± 95.08	45.21 ± 10.75	118.92 ± 46.79	111.76 ± 36.44	131.86 ± 17.46	79.91 ± 12.22	8.83 ± 5.84	678.66± 258.64	9.58 ± 3.79
GC	24.55 ± 3.52	192.25 ± 37.25	140.93 ± 80.08	48.07 ± 18.31	112.05 ± 34.83	117.05 ± 41.53	128.96 ± 15.82	80.71 ± 11.11	8.80 ± 7.18	681.88± 271.16	10.19 ± 4.60
CC	25.44 ± 4.05	193.55 ± 36.28	152.79 ± 107.61	47.28 ± 10.51	115.06 ± 26.77	118.14 ± 24.38	140.17 ± 18.83	82.10 ± 12.02	8.09 ± 5.09	689.37± 269.45	10.42 ± 4.70
*p*-value	0.236	0.975	0.64	0.423	0.59	0.349	0.005	0.574	0.834	0.995	0.28
rs12826786 C>T											
CC	24.05 ± 3.32	192.55 ± 37.82	150.01 ± 93.69	45.62 ± 11.74	117.68 ± 46.72	114.27 ± 40.69	131.57 ± 17.64	80.10 ± 12.03	8.49 ± 5.64	676.39± 257.63	9.82 ± 4.14
CT	25.12 ± 3.56	192.58 ± 36.64	142.98 ± 88.14	47.60 ± 18.03	115.31 ± 28.00	113.29 ± 30.45	131.03 ± 16.36	80.90 ± 11.55	9.47 ± 7.50	710.68± 290.40	9.80 ± 4.05
TT	23.12 ± 1.95	176.10 ± 25.63	109.80 ± 44.98	50.13 ± 9.50	96.57 ± 15.22	112.40 ± 21.86	134.00 ± 16.63	79.60 ± 10.52	7.57 ± 5.39	643.20± 286.57	10.84 ± 5.40
*p*-value	0.042	0.387	0.334	0.526	0.295	0.963	0.858	0.806	0.291	0.438	0.741
rs874945 C>T											
CC	24.09 ± 3.37	192.18 ± 37.24	148.99 ± 93.61	45.53 ± 11.86	117.98 ± 46.91	114.09 ± 39.70	131.46 ± 17.93	80.27 ± 12.13	8.45 ± 5.71	675.71± 257.23	9.90 ± 4.44
CT	24.97 ± 3.49	193.91 ± 37.19	143.29 ± 87.18	48.16 ± 17.67	114.20 ± 29.67	112.31 ± 33.68	131.02 ± 16.06	80.62 ± 11.56	9.59 ± 7.34	706.34± 286.03	9.72 ± 3.49
TT	23.49 ± 2.72	179.33 ± 36.91	142.72 ± 89.13	46.35 ± 10.13	106.20 ± 26.71	123.17 ± 29.98	135.00 ± 14.75	79.06 ± 9.65	7.32 ± 4.72	687.78± 304.89	9.90 ± 4.22
*p*-value	0.102	0.299	0.83	0.515	0.634	0.514	0.655	0.864	0.144	0.569	0.917

ANOVA, analysis of variance; HDL cholesterol, high-density lipoprotein cholesterol; LDL cholesterol, low-density lipoprotein cholesterol; BMI, body mass index; FBS, fasting blood sugar; SBP, systolic blood pressure; DBP, diastolic blood pressure; SD, standard deviation.

**Table 8:** Clinical variables in CAD patients stratified by *HOTAIR* polymorphisms status by ANOVA.

Genotypes	BMI (kg/m^2^)	Total Cholesterol (mg/dL)	Triglyceride (mg/dL)	HDL Cholsterol (mg/dL)	LDL Cholesterol (mg/dL)	FBS (mg/dL)	SBP (mmHg)	DBP (mmHg)	Folate (mg/mL)	Vitamin B12 (pg/mL)	Homocysteine (mmol/L)
	Mean ± SD (434)	Mean ± SD (439)	Mean ± SD (439)	Mean ± SD (437)	Mean ± SD (425)	Mean ± SD (430)	Mean ± SD (442)	Mean ± SD (442)	Mean ± SD (404)	Mean ± SD (90)	Mean ± SD (422)
rs4759314 A>G											
AA	25.10 ± 3.36	188.08 ± 48.02	158.24 ± 91.66	43.75 ± 11.20	113.32 ± 41.58	140.92 ± 63.42	127.73 ± 20.35	79.06 ± 12.75	8.72 ± 9.95	707.46± 305.54	9.57 ± 4.54
AG	25.06 ± 3.05	179.90 ± 34.72	157.95 ± 79.00	42.98 ± 11.50	103.19 ± 34.00	142.08 ± 57.08	127.95 ± 23.12	75.63 ± 13.26	9.40 ± 7.47	557.00± 299.12	10.43 ± 5.89
GG	0.00 ± 0.00	0.00 ± 0.00	0.00 ± 0.00	0.00 ± 0.00	0.00 ± 0.00	0.00 ± 0.00	0.00 ± 0.00	0.00 ± 0.00	0.00 ± 0.00	0.00± 0.00	0.00 ± 0.00
*p*-value	0.951	0.306	0.985	0.689	0.162	0.914	0.951	0.114	0.699	0.188	0.293
rs1899663 G>T											
GG	25.10 ± 3.41	185.49 ± 42.46	151.41 ± 87.64	43.00 ± 10.99	112.38 ± 37.35	142.95 ± 63.05	128.16 ± 20.94	78.86 ± 13.00	8.85 ± 7.85	717.00± 308.06	9.50 ± 4.49
GT	25.08 ± 3.30	190.99 ± 50.39	168.58 ± 93.53	44.65 ± 11.71	112.35 ± 43.50	134.70 ± 59.86	128.37 ± 20.03	79.31 ± 12.73	9.04 ± 12.69	630.27± 287.26	10.01 ± 5.07
TT	25.04 ± 2.76	183.85 ± 66.15	161.89 ± 97.49	44.47 ± 10.09	114.39 ± 58.51	161.58 ± 75.54	120.00 ± 19.39	74.62 ± 11.04	6.37 ± 4.32	832.50± 576.29	8.90 ± 3.80
*p*-value	0.994	0.476	0.169	0.327	0.971	0.112	0.140	0.221	0.483	0.399	0.403
rs920778 T>C											
TT	25.02 ± 3.44	186.89 ± 44.81	149.96 ± 82.43	42.78 ± 10.79	114.29 ± 39.73	143.74 ± 64.77	127.64 ± 21.10	79.06 ± 13.06	8.76 ± 7.78	606.50± 424.48	9.41 ± 4.48
TC	25.18 ± 3.32	190.00 ± 50.41	171.17 ± 101.05	44.93 ± 11.56	112.12 ± 43.96	136.45 ± 59.49	129.20 ± 19.91	79.10 ± 12.65	9.09 ± 12.28	633.90± 290.25	9.89 ± 4.67
CC	25.17 ± 2.42	173.85 ± 40.87	141.15 ± 71.54	42.85 ± 11.95	99.52 ± 29.24	149.15 ± 68.69	118.89 ± 18.88	74.07 ± 11.18	6.91 ± 4.80	736.49± 305.82	10.04 ± 6.21
*p*-value	0.883	0.245	0.037	0.145	0.219	0.406	0.051	0.145	0.582	0.290	0.550
rs7958904 G>C											
GG	25.13 ± 3.41	185.74 ± 45.08	151.22 ± 81.47	42.58 ± 10.69	113.72 ± 39.30	143.46 ± 63.23	127.70 ± 21.40	78.91 ± 12.98	8.76 ± 7.79	606.50± 424.48	9.47 ± 4.40
GC	25.00 ± 3.33	192.07 ± 50.62	170.82 ± 104.04	45.28 ± 11.71	112.43 ± 44.85	136.39 ± 61.73	128.87 ± 18.63	79.13 ± 12.39	9.14 ± 12.62	635.74± 290.14	9.91 ± 4.80
CC	25.32 ± 2.73	174.45 ± 39.70	145.55 ± 74.08	43.75 ± 11.90	102.81 ± 32.49	147.32 ± 66.53	121.72 ± 23.61	75.52 ± 13.78	6.93 ± 4.60	735.37± 306.26	9.65 ± 6.10
*p*-value	0.859	0.127	0.073	0.058	0.403	0.466	0.225	0.364	0.555	0.308	0.648
rs12826786 C>T											
CC	25.07 ± 3.42	184.57 ± 42.13	151.50 ± 86.75	42.63 ± 10.77	112.11 ± 36.78	142.90 ± 63.31	127.73 ± 21.39	78.52 ± 13.00	8.92 ± 7.83	717.67± 307.57	9.53 ± 4.45
CT	25.02 ± 3.26	193.75 ± 54.49	169.48 ± 97.85	45.61 ± 11.92	114.59 ± 48.32	137.51 ± 62.54	127.95 ± 19.63	79.38 ± 12.78	8.89 ± 12.66	643.32± 303.21	9.94 ± 5.07
TT	26.32 ± 2.28	169.33 ± 36.00	157.27 ± 65.08	41.71 ± 8.62	97.47 ± 25.16	146.00 ± 60.00	126.00 ± 16.39	76.67 ± 9.76	5.45 ± 2.78	0.00± 0.00	8.77 ± 4.11
*p*-value	0.345	0.199	0.141	0.023	0.426	0.667	0.940	0.648	0.405	0.294	0.525
rs874945 C>T											
CC	25.07 ± 3.41	185.72 ± 42.10	151.47 ± 87.40	42.56 ± 10.83	113.12 ± 36.76	143.30 ± 64.15	128.32 ± 21.39	78.82 ± 13.01	8.73 ± 7.65	717.67± 307.57	9.51 ± 4.47
CT	25.04 ± 3.31	192.32 ± 54.80	169.82 ± 97.07	45.54 ± 11.68	113.43 ± 48.46	135.48 ± 58.84	127.50 ± 19.70	79.02 ± 12.88	9.17 ± 12.83	628.77± 287.97	9.94 ± 5.05
TT	25.78 ± 2.46	168.55 ± 32.85	150.75 ± 66.38	42.85 ± 10.75	97.35 ± 23.01	157.95 ± 76.01	122.50 ± 16.50	76.00 ± 9.40	6.42 ± 4.41	832.50± 576.29	9.16 ± 4.15
*p*-value	0.637	0.193	0.121	0.029	0.208	0.228	0.468	0.606	0.509	0.385	0.593

ANOVA, analysis of variance; HDL cholesterol, high-density lipoprotein cholesterol; LDL cholesterol, low-density lipoprotein cholesterol; BMI, body mass index; FBS, fasting blood sugar; SBP, systolic blood pressure; DBP, diastolic blood pressure; SD, standard deviation.

## Data Availability

Data sharing not applicable.

## Supplementary Materials

The following are available online at https://www.mdpi.com/article/10.3390/jpm11050375/s1, Table S1: Details of HOTAIR polymorphisms for PCR-RFLP analysis.
