# Supplementary material for: Association between HOTAIR lncRNA Polymorphisms and Coronary Artery Disease Susceptibility
Source: J Pers Med. 2021 May 4;11(5):375. doi: 10.3390/jpm11050375 (PMC8147832; doi:10.3390/jpm11050375)
Supplement: Supplementary file 1 [file jpm-11-00375-s001.zip › jpm-1177231-SI.pdf]

Table S1. Details of *HOTAIR* polymorphisms for PCR-RFLP analysis

**rs number**		**primer**	**Restriction enzyme**
rs1899663	forward	5'- TTT TCC AGT TGA GGA GGG TGG A -3'	*Hph*I
	reverse	5'- CTA ATG GCA AGG GAA GGG AAG G -3'	
rs4759314	forward	5'- ACC CAA AAC CAT TTC CTG AGA G -3'	*Alu*I
	reverse	5'- TTC AGG TTT TAT TAA CTT GCA TCA GC -3'	
